# The relationship between religion and livelihood activities of women: Empirical evidence from the Yilo and Lower Manya Krobo Districts of Eastern Ghana

**DOI:** 10.1016/j.ribaf.2020.101311

**Published:** 2021-01

**Authors:** Emmanuel Ayifah, Aylit Tina Romm, Umakrishnan Kollamparambil

**Affiliations:** a School of Economics and Finance, University of the Witwatersrand, Johannesburg, South Africa and SEND Ghana, A28 Regimanuel Estate, Nungua Barrier, Sakumano, Accra, Ghana; b School of Economics and Finance, University of the Witwatersrand, Johannesburg, South Africa

**Keywords:** Religion, Risk behavior, Women, Livelihood activities, Ghana

## Abstract

The study examines the effect of religious affiliation on livelihood activity choice amongst a sample of 1209 women in the Yilo and Lower Manya Krobo Districts of Ghana. We attempt to disentangle the effect religion would have through its effect on risk preferences, from the effect it would have due to creation of social capital or the effect of clustering due to social identity. We find evidence that there is a strong positive social capital or social identity effect of being Catholic, Pentecostal or Protestant when it comes to farming. This effect is also positive for Protestant and Catholic women for wage employment. This social capital and social identity effect is significantly negative for Catholic, Pentecostal and Protestant women in the case of petty retailing. Women who are willing to take risk are more likely to be in farming and less likely to be wage employed than risk averse women. However, the effect of risk aversion as a result of being Catholic, Protestant or Pentecostal only seems to have a negative effect in the case of farming. The risk aversion effect does not appear to act through religious affiliation when it comes to wage employment. It is recommended that any policy intervention aimed at enhancing livelihood activities of women in this area should take into consideration any relative advantage of having a specific religious affiliation. Such policies should take cognizance of both clustering and networking effects resulting from belonging to a religious group. Risk profiles of women are also important when implementing such policies. Other important considerations are age, household headship, education, dependency ratio, household farm ownership and household enterprise ownership.

## Graphical abstract

**Figure:**
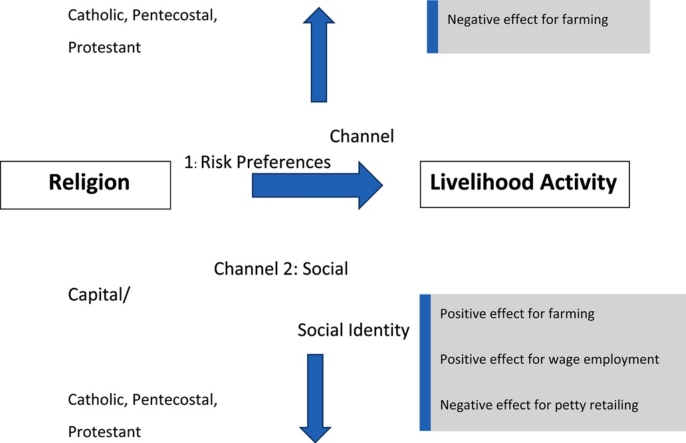

## 1 Introduction

While historically men have been seen as the breadwinners in Ghana, the status quo is changing and women are becoming important economic actors in the household (Amu, 2005; Hesselberg and Yaro, 2006; Wrigley-Asante, 2011). In Ghana, in particular, women contribute greatly to household food security (IFAD, 1998; Kunze and Drafor, 2003; Hesselberg and Yaro, 2006; Wrigley-Asante, 2011;). Women increase their contribution to household food security by either growing food or by earning income to purchase food (IFAD, 1998). Thus, how women in Ghana choose a livelihood activity is important. In this paper, we investigate the effect of a woman’s religious affiliation on her choice of livelihood activity in the Yilo and lower Manya Krobo districts of Ghana.

Studies based on rural communities in Africa have identified many factors that determine livelihood choice. Such factors include: age (Babulo et al., 2008; Wanyama et al., 2010; Dary and Kuunibe, 2012; Tran et al., 2014), gender (Babulo et al., 2008; Wanyama et al., 2010; Dary and Kuunibe, 2012; Tran et al., 2014), marital status (Dary and Kuunibe, 2012) and education (Babulo et al., 2008; Dary and Kuunibe, 2012; Shehu and Abubakar, 2015). Other factors include: household size (Babulo et al., 2008; Shehu and Abubakar, 2015), credit access (Brown et al., 2006; Babulo et al., 2008; Wanyama et al., 2010; Kuwornu et al., 2014; Shehu and Abubakar, 2015), remittances (Brown et al., 2006), access and distance to market (Babulo et al., 2008; Wanyama et al., 2010; Shehu and Abubakar, 2015), location (Brown et al., 2006; Dary and Kuunibe, 2012; Shehu and Abubakar, 2015), farm ownership, farm size, farm assets as well as farm loss (Babulo et al., 2008; Wanyama et al., 2010; Tran et al., 2014; Kuwornu et al., 2014) and access to grazing land (Babulo et al., 2008). In addition, some studies looked at factors such as access to electricity and mobile phone services (Shehu and Abubakar, 2015) and social networks (Dary and Kuunibe, 2012; Shehu and Abubakar, 2015).

Research on the effect of religion on livelihood choice is very sparse. There has been a minimal amount in developed countries. Greeley (1963) and Sigalow (2012) for the US are examples of some of the few studies that have looked at this. In the rural African context, we are not aware of any authors that have specifically investigated this issue^1^ .

While the importance of social capital and social networks in choosing a livelihood activity has been documented in the literature (Scoones, 1998 and in the rural African context Dary and Kunnibe, 2012 and Shehu and Abubakar, 2015), the importance of religion as a specific type of social capital, and thus its particular role in choice of livelihood activity, has not been investigated. Social capital is essential for facilitating and sustaining diverse income portfolios and access to opportunities and resources to individual households (Berry, 1989; Hart, 1995; Bryceson, 1996). Religious organizations are recognized in the social capital literature as producers and facilitators of social capital (Yeary, 2012; Smidt, 2006). In addition, based on the ‘social identity theory’ and ‘identity theory’ in social Psychology (Tajfel, 1972b; Stets and Burke, 2000; Greenfield and Marks, 2007)^2^, it can be argued that having common religious beliefs can encourage individuals to group together. Such grouping, we hypothesize, may cause members of the same religion to engage in the same livelihood activities.

Religious affiliation could have an additional effect on livelihood activity choice, through its effect on risk behavior. Again, based on the ‘social identity theory’ and ‘identity theory’ in social Psychology, it can be argued that religious beliefs can influence individual preferences in general, and that individuals belonging to a religious group behave in a particular way (Inzlicht et al., 2009). Consequently, religion may lead to heterogeneity in individual attitudes, preferences, and traits. Thus, risk behavior of an individual may differ based on his or her religious affiliation. Studies done in various country contexts c.f., Halek and Eisenhauer (2001); Hilary and Hui (2009); Benjamin et al. (2010, 2013) for the US; Dohmen et al. (2011); Weber (2013) for Germany; Renneboog and Spaenjers (2012) for the Netherlands; Ayifah et al. (2020) for Ghana find religious doctrines to influence individual attitudes and preferences, including diet, marriage, investment, and risk-seeking propensity among others. The results of Ayifah et al. (2020) showed, using the same sample/data as the current study, that religion was a significant determinant of the Ghanaian Woman’s propensity to take risk. In particular, religious women were far more risk averse than non-religious women.

Numerous studies have looked at the relationship between risk attitudes and occupation/livelihood choices, and have found that risk averse individuals prefer to be in jobs that involve less income risk. Bellante and Link (1981) showed that risk averse individuals in the United States were more likely to work in the public sector than they were to work in the private sector. Cramer et al. (2002) showed that risk averse individuals in The Netherlands were less likely to be self-employed. Bonin et al. (2007) found that risk averse individuals in Germany were more likely to work in occupations with low earning risk. For the case of Ethiopia, Bezabih et al. (2010) found that individuals with risk neutral preferences were more likely to engage in off farm activities. Cho (2012) looked at the entrepreneurship decisions of men and women separately for the case of the United States. They showed that while risk averse men were more likely to choose paid employment than to be self-employed, there was no significant effect of risk preferences on women’s entrepreneurship decisions. Le et al. (2014) showed that risk-seeking individuals in Australia had a higher probability of being employed in more prestigious, high paying professional and administrative occupations. One study looking at the relationship between risk preferences and occupational choice for the case of Ghana is the study by Falco (2014). Looking at urban Ghanaians, he looks at the effect of risk preferences on being employed in the formal and informal sectors. He finds that risk averse individuals are more likely to be employed in the formal sector compared to the informal sector.

Our current study contributes to the literature in the following ways. We look at the effect of religion on livelihood choice. We have found very little literature investigating this issue in general and certainly in the African context. In particular, we feel we are the first to look at this issue for women, and specifically for African Women. The importance of social capital and networks facilitated by religious affiliations might be especially important for women. In addition, the effect of religion on livelihood choice through its effect on risk behavior has not to our knowledge been investigated anywhere before. Thus, we contribute by disentangling the separate effects religion might have on livelihood choice: The first effect being that of social capital and “social identity”, and the second effect being the risk behavior effect.

The current study therefore seeks to establish empirically a relationship between religious affiliation and choice of livelihood activity, using data derived from a sample of 1209 women in the Yilo and Lower Manya Krobo districts of Ghana. The five main livelihood activity categories considered are farming, petty retailing, vocation, paid employment and no livelihood activity. The religious affiliations considered are Protestant, Pentecostal, Catholic, Muslim, traditional religion and no religion.

We proceed with the paper as follows: Section 2 presents the data and methodology, Section 3 presents some descriptive statistics, Section 4 shows the regression results, and we conclude with Section 5.

## 2 Methodology and data

### 2.1 Methodology

We use data on 1209 women from the Socio-economic Studies (SES) arm of the International Lipid-Based Nutrient Supplement Research Project in Ghana (iLiNS DYAD-G SES). The iLiNS DYAD-G-SES study is a collaborative research between the University of Ghana and the University of California, Davis. The iLiNS DYAD-G-SES study protocol was approved by the Institutional Review Board (IRB) of the University of California, Davis, USA and the Noguchi Memorial Institute of Medical Research, University of Ghana IRB. The iLiNS project study area is along a stretch of busy commercial corridor in the Lower Manya Krobo and Yilo Krobo districts. As part of the eligibility criteria into the iLiNS study; women should be at least 18 years of age, resident in the Lower Manya Krobo or Yilo Krobo districts throughout the study period and be prepared to sign an informed consent (with approval from her husband or household head). For this study, we use the Household Socio-economic Characteristics data and Risk Aversion data from the iLiNS DYAD-G SES study. The household socio-economic characteristics data contain information on the main livelihood activities of our sampled women 12 months prior to the survey. It also contains data on the religious affiliation of these women. The experimental risk behavior data was collected using experimental risk behavior games with real monetary pay-offs.

For a woman faced with multinomial livelihood activity choices, we hypothesise that there is a relationship between her religious affiliation and livelihood activity choice and therefore estimate the Eq. (7) below:

(7) LAij=β+γReligionik+δXi+μi;μi∼∼Normal0,1

Where, $LA_{ij}$ represents multiple unordered livelihood activities of the woman including farming, retailing, vocation, paid employment or no livelihood activity (unemployed). *Religion* is a set of dummy variables for the religious affiliation of the woman including, Pentecostal, Protestant, Catholic, Muslim, traditional religion and no religious affiliation. X_i_ is a vector of individual and household control variables (including risk preferences) that are likely to also affect choice of livelihood activity. β is the intercept, γ and δ are the parameters, $\mu_{i}$ is a normally distributed random error term with mean zero, summarising all unobserved factors that affect livelihood activity.

In our unordered multinomial model, the coefficients have qualitative effects on L *A*. Therefore, quantitative predictions are made only based on the marginal effects of the regressors, since the parameters of multinomial models are generally not directly interpretable. Interest therefore lies in how the probabilities of the alternative livelihood activities (LA) change with different religious affiliations (the base category is individuals who have no religious affiliation. Hence, we compute the marginal effects of individual *i* belonging to a specific religious group (*k*) relative to the base category, on the probability that an individual *i* chooses alternative LAj as:

(8) $$\frac{\partial P_{ij}}{\partial {religion}_{ik}}=P_{ij}(\gamma_{i}-\bar{\gamma_{i})}$$

Where; $\bar{\gamma_{i}}=\sum_{i}P_{ij}\gamma_{i}$ is a probability-weighted average of the parameter, $\gamma_{i}$. The marginal effects vary with the point of evaluation of *religion_k_*, because the probability of the ith individual choosing livelihood activity j varies with *religion_k_*. For *religion_k_*, the marginal effect is positive if $\gamma_{j}>\bar{\gamma_{i}}$.

A major concern of the multinomial logit (Mlogit) estimation technique is the relationship between probabilities; that is the popular assumption of Independence of Irrelevant Alternatives (IIA). Hence, we use the Hausman and McFadden (1984) decision rule in our analysis.

### 2.2 Variable definitions

With respect to our outcome variable - livelihood activities of women - we identified and grouped livelihoods into five main livelihood activities namely: farming, petty retailing, vocation, paid employment and no livelihood activity (unemployed). Farming involves subsistence food crop cultivation. Petty retailers are those whose livelihood activities involve mainly buying and selling small consumable and non-durable items. Vocation as a livelihood activity primarily involves self-employed professional or vocational work such as being a seamstress or a beautician (hairdressing, pedicure and manicure); and wage employed are those in either formal or informal livelihoods (nurses, teachers, laborers, factory hand etc.). Lastly, ‘no livelihood’ constitutes unemployed women who do not engage in any livelihood activity.

With respect to our Religion variable, we use dummy variables representing the religious affiliation of the woman ( = 1 if a member of a religious group and 0 otherwise). The religious affiliation variables used include Pentecostal, Protestants, Catholics, Muslims, Traditional religion and No religion.

Risk behaviour is measure of a woman’s Willingness to Take Risk (WTR) in a risk aversion game with real monetary pay-offs^3^ . As indicated earlier, our risk behaviour variable is binary, where; ${WTR}_{i}$ = 1 if a woman is willing to take risk; otherwise, 0.

Various studies have identified many possible factors that influence livelihood activity choice. Most common factors in the literature seen to affect the choice of livelihood activity are age, marital status and education (Babulo et al., 2008; Dary and Kuunibe, 2012; Tran et al. 2014; Shehu and Abubakar, 2015). Others factors include household size, credit access, remittances access and distance to market, location, farm ownership, farm size, farm assets as well as farm loss and access to grazing land (Babulo et al., 2008; Tran et al., 2014; Shehu and Abubakar, 2015; Brown et al., 2006; Wanyama et al., 2010; Kuwornu et al., 2014; Dary and Kuunibe, 2012; Tran et al., 2014). Some studies have also looked at factors such as access to electricity and mobile phone services (Shehu and Abubakar, 2015) and social networks (Dary and Kuunibe, 2012; Shehu and Abubakar, 2015). Accordingly, we have included the most relevant in the analysis. Individual and household characteristics used as control variables include age, marital status, education, general social capital, household headship, dependency ratio, household size, household farm ownership and household enterprise ownership.

*Age:* Age of the study woman is measured in completed years as a continuous variable.

*Marital Status:* Study participants were asked to indicate whether they are actually married, in loose union, divorce, separated, or never married. Marital status is therefore measured using a dummy variable; with 1, representing women who are legally married through ordinance (civil) or customary (traditional) recognition and 0 if not married (including those in loose or informal unions, divorce, separated, or never married.

*Education:* Education of our study women is measured by a dummy variable; 1 representing educated and 0 if the woman is not educated.

*Household head:* We use a dummy variable to represent household headship (1 if the woman is a household head, 0 otherwise).

*General Social capital*^4^ : Social capital may serve as a means of social network or support, fallback strategies through which people get access to various kinds of livelihoods. In this study, while we acknowledge that belonging to a religion is a form of social capital, we control also for all kinds of social capital with the variable *General social capital.* It is measured as a continuous variable, indicating the total number of groups or associations to which a woman belongs.

*Per capita income:* Is a continuous variable measured as total household monthly income per the total number of household members.

*Household Farm:* This is a dummy variable for household ownership of a farm, where, 1 if the household owns a farm, otherwise 0.

*Household Enterprise:* We use a dummy variable to represent household ownership of an enterprise (1 if the respondent’s household owns an enterprise, 0 otherwise).

## 3 Descriptive statistics

Table 1 shows some summary statistics. We identify five main livelihood activities of women. These are farming, retailing, vocation, paid employment or no livelihood activity (unemployed). Our study women are on average 27 years of age, and about 98.8 percent are married. Approximately 91 percent are educated; and less than one-fifth (14 percent) are household heads. In terms of religious affiliation of study participants, more than half of the women (67 percent) are Pentecostals, followed by Protestants (18 percent) and Catholics (9 percent). Muslims and traditional religious constitute 3 percent each, with the non-religious forming just 1 percent. On average, there are four people in a household, with an average per-capita income of approximately GH¢ (GHc84, $51). About 22 percent of households own farms and as much as 86 percent own household enterprises/businesses.

**Table 1:** Summary Statistics.

	Variable	Definition	Mean	Std. Dev.	Min	Max
*Livelihood Activities	No livelihood	= 1 No livelihood activity, otherwise 0	.2002	.4003	0	1
	Farming	= 1 farmer, otherwise 0	.0132	.1143	0	1
	Petty Retailing	= 1 petty retailer, otherwise 0	.5227	.4997	0	1
	Vocation	= 1 vocation, otherwise 0	.1663	.3725	0	1
	Wage Employed	= 1 wage employed, otherwise 0	.0968	.2957	0	1
**Risk Behavior**	Willing to take risk	= 1 willing to take risk, otherwise 0	.9586	.1992	0	1
**Individual characteristics**	Age in years	Age in years as a continuous variable	26.8247	5.5079	18	44
	Marital Status	= 1 married, otherwise 0	.9884	.1070	0	1
	Education	= 1 educated, otherwise 0	.9065	.2912	0	1
	General Social Capital	Number of groups or associations a woman belongs to	.6683	.7335	0	5
	Household headship	= 1 if household head, otherwise 0	.1422	.3494	0	1
	Religious Affiliation Pentecostal Protestant Catholic Muslim Traditional No religion	= 1penticostal, otherwise 0 = 1 protestant, otherwise 0 = 1 catholic, otherwise 0 = 1 Muslim, otherwise 0 = 1 traditional religion, otherwise 0 = 1 No religion, otherwise 0	.6674 .1811 .0860 .0248 .0322 .0083	.4713 .3852 .2805 .1556 .1768 .0906	0 0 0 0 0 0	1 1 1 1 1 1
**Household characteristics**	Household Size	Total number of household members	3.9148	2.1369	1	16
	Per capita income	Per capita income in Gh¢**	83.9122	96.0695	0	850
	Household farm	= 1 if household owns a farmland, otherwise 0	.2159	.4116	0	1
	Household enterprise	= 1 if household operates an enterprise, otherwise 0	.8379	.3687	0	1

The majority (52 percent) identified petty retailing as their main livelihood activity, while 17 percent and 10 percent identified vocation and paid employment respectively as their livelihoods activities. Those who reported farming as their main livelihood activity constituted just a little over 1 percent. This result is not surprising because the study area is along a long stretch of road with a busy commercial activity, and as with most towns along busy commercial corridors in Ghana, non-farm livelihood activities contribute significantly to livelihood activities of residents of such areas. It is also worth acknowledging that a sizeable number of the women in our sample indicated they are not engaged in any livelihood activity. In terms of individual risk behavior, approximately 4.2 percent of our study women were not willing to take risk and chose not to play the risk aversion game at all. The remaining 95.8 percent were willing to take risk.

Table 2 shows for each religious affiliation, the proportion of women engaging in different likelihood activities. It is evident that non-religious women are more likely than religious women to have no livelihood activity. The only livelihood activity that women with no religious affiliation engage in at all is petty retailing. It is also evident that Muslim women, and those affiliated with a traditional religion do not engage in farming at all as a livelihood activity. We also see that petty retailing is the most popular livelihood activity amongst all religions (including non-religious), and that farming is the least popular livelihood activity amongst all religious groups (including non-religious).

**Table 2:** Religious Affiliation by Livelihood Activities.

Religion (%)	Livelihood Activities (%)					Total (%)
	No Livelihood	Farming	Petty Retailing	Vocation	Paid employment	
no religion	40.00	0.00	60.00	0.00	0.00	**0.83**
Pentecostal	19.45	1.36	54.52	16.48	8.18	**66.75**
protestant	20.09	1.83	44.75	17.35	15.98	**18.11**
Catholic	18.27	0.96	47.12	21.15	12.50	**8.60**
Muslim	20.00	0.00	60.00	10.00	10.00	**2.48**
traditional	30.77	0.00	53.85	12.82	2.56	**3.23**
**Total**	**20.02**	**1.32**	**52.27**	**16.63**	**9.76**	**100.00**
likelihood-ratio chi2(20) = 31.5861 Pr = 0.048						

Table 3 shows the percentage of women in each livelihood activity that are willing to take risk.

**Table 3:** Risk Behavior by Livelihood Activity.

Livelihood Activities	Risk behaviour		Total (%)
	Not willing to take risk (%)	Willing to take risk (%)	
No Livelihood	2.48	97.52	20.02
Farming	0	100	1.32
Petty Retailing	4.59	95.41	52.27
Vocation	3.48	96.52	16.63
Paid employment	6.78	93.22	9.76
**Total**	**4.14**	**95.86**	**100**
likelihood-ratio chi2(4) = 5.5969 Pr = 0.231			

Farming attracts the most risk takers, while paid employment attracts the least amount of risk takers.

Table 4 shows the proportions willing and not willing to take risk (engage or not engage in the gamble in the game) for each religious affiliation. Clearly every women in our sample who has no religious affiliation, is willing to take risk. The Religious group with the least percentage of women willing to take risk is Muslims.

**Table 4:** Risk Behavior by Religious Affiliation.

Religious Affiliation	Risk behaviour		Total (%)
	Not willing to take risk (%)	Willing to take risk (%)	
No Religion	0	100	0.83
Pentecostal	4.46	95.54	66.75
Catholic	3.84	96.16	8.60
Protestant	3.20	96.80	18.11
Muslim	6.67	93.33	2.48
Traditional Religion	2.56	97.44	100

## 4 Econometric results and discussion

### 4.1 Results

Our aim is to investigate the effect of religion on livelihood activity choice by disentangling the two possible channels through which the effect of religion might operate: 1) The effect of social capital, networking and identity resulting from being affiliated to a specific religion; 2) The effect of risk aversion resulting from belonging to a specific religious group.

To ensure identification of our model we set no livelihood activity as the reference category, and therefore estimate a model for the other livelihood activity choices (farming, petty retailing, vocation and paid employment) relative to no livelihood activity. In terms of the religion variable, we use *no religion* as the base category. We have dummy variables for *Pentecostal, Protestant, Catholic,* and *Muslim* and look at the effect of belonging to each of these categories relative to having *no religion*. We do not include *traditional religion* in our regression analysis because unfortunately including this variable leads to non-convergence. For ease of interpretation, we present the marginal effects with respect to no livelihood activity.

Tables 5 presents the results, shown as marginal effects of the mlogit regression that includes religion as an explanatory variable, without including the risk behavior variable. This shows the overall effect of religion on livelihood choice- both the effect it has through religious social capital and identity, and the effect it has through risk behavior.

**Table 5:** MLOGIT JUST WITH RELIGION (NO WTR). Multinomial Logit Marginal Effect (No Livelihood as Benchmark).

	VARIABLES	Farming	Petty Retailing	Vocation	Wage Employed
**Religion**	Pentecostal	0.1403***	−0.1634**	0.0307	0.0955
		(0.0352)	(0.0794)	(0.0624)	(0.0749)
	Catholic	0.1304***	−0.2320***	0.0687	0.1210
		(0.0328)	(0.0877)	(0.0688)	(0.0767)
	Protestant	0.1404***	−0.2161***	0.0472	0.1320*
		(0.0354)	(0.0832)	(0.0650)	(0.0759)
	Muslim	−0.0074	−0.0822	−0.0136	0.1128
		(0.0104)	(0.1141)	(0.1020)	(0.0918)
**Individual characteristics**	Age	0.0006	0.0160***	0.0032*	0.0045***
		(0.0005)	(0.0026)	(0.0019)	(0.0014)
	Married	0.00522	−0.0180	0.0261	0.0211
		(0.0076)	(0.0296)	(0.0238)	(0.0158)
	Education	−0.0002	−0.1412***	0.0646	0.0347
		(0.0087)	(0.0467)	(0.0434)	(0.0324)
	General Social capital	−0.0008	−0.0269	0.0225	0.0052
		(0.0029)	(0.0175)	(0.0140)	(0.0093)
	Household head	0.0176**	0.0768*	0.0774***	−0.0058
		(0.0083)	(0.0412)	(0.0295)	(0.0223)
**Household characteristics**	Dependency ratio	0.0008	0.0918***	−0.0840***	−0.0049
		(0.0038)	(0.0220)	(0.0217)	(0.0122)
	Per-capita income	−0.0001	0.0004**	1.15e-05	0.0002***
		(7.19e-05)	(0.0002)	(0.0001)	(6.14e-05)
	Household farm	0.0358***	−0.0124	−0.0107	−1.72e-05
		(0.0104)	(0.0323)	(0.0263)	(0.0197)
	Household enterprise	−0.0179***	0.3334***	0.1230**	−0.1672***
		(0.0054)	(0.0542)	(0.0492)	(0.0144)
	Observations	1209	1209	1209	1209

Note: Robust standard errors in parentheses; *** p < 0.01, ** p < 0.05, * p < 0.1.

The results show that relative to non-religious women, the likelihood of Pentecostal women being in farming compared to having no livelihood activity is 14.03 percentage points higher, and 16.34 percentage points lower for being in petty retailing, compared to having no livelihood activity. For Catholic women relative to non-religious women, the likelihood of being in farming relative to having no livelihood is 13.04-percentage points higher and 23.02 percentage points lower for petty retailing compared to having no livelihood. For protestant women relative to non-religious women, the likelihood of being in farming relative to not having a livelihood is 14.04 percentage points higher, it is 21.61 percentage points lower for petty retailing, and 13.20 percentage points higher for being wage employed. Being Muslim relative to belonging to no religion, does not significantly change the likelihood of having any particular livelihood activity relative to having no livelihood activity.

The results show a positive and statistically significant relationship between a woman’s age and petty retailing, vocation and paid employment relative to no livelihood activity. This result is expected because as women age they assume more responsibilities in the household, hence they prefer to engage in some form of livelihood to support their households relative to not engaging in any livelihood activity. However, age does not significantly change the probability of engaging in farming relative to having no livelihood activity.

Being married has no significant effect on engaging in any livelihood activity (farming, petty retailing, vocation and paid employment) relative to having no livelihood activity. The education variable is statistically significant only for the choice of petty retailing as a livelihood activity. With a negative statistically significant marginal effect (p < 0.01), the result shows that if a woman is educated she is 14.12 percentage points less likely to engage in petty retailing relative to having no livelihood activity. This is an indication that the educated would rather not engage in any livelihood activity than to engage in petty retailing, at least until they get the chance to work in formal or wage employed activities. This is particularly so in the context of the study area and other parts of Ghana where engaging in petty retailing as a means of livelihood is considered to be the preserve of the uneducated and unskilled.

As expected, we see a positive, statistically significant relationship between household headship and farming, petty retailing and vocation. Being a household head comes with the responsibility of caring for the household members, hence it is not surprising that household heads would choose to engage in any livelihood activity just to enable them take care of household needs rather than with no livelihood activity. Being a household head increases a woman’s probability of being in farming by 1.76 percentage points, of being in retailing by 7.68 percentage points and of having a vocation by 7.74 percentage points.

In terms of household characteristics, a unit increase in the dependency ratio significantly increases the probability of choosing petty retailing by 9.18 percentage points and decreases the probability of engaging in a vocation by 8.4 percentage points, relative to having no livelihood activity. Compared with petty retailing, having a vocation involves relatively higher financial and logistical investments. Hence, it makes sense that an increase in dependency ratio would increase the probability of engaging in petty retailing, but decrease the probability of choosing vocation relative to no livelihood activity.

Additionally, per-capita household income is significantly associated with being involved in petty retailing, as well as paid employment, but the effect is very marginal in both cases (less than 1 percentage point increase), relative to the benchmark category. All things being equal, if a woman’s household owns a farm, it is expected that she will choose farming as a livelihood activity relative to no livelihood activity, thus the significant positive effect.

As expected, relative to no livelihood activity, household enterprise ownership is positive and significantly related to the choice of petty retailing and vocation and; negatively related to farming and paid employment. If a woman’s household owns an enterprise she is 33.34 percentage points more likely to engage in petty retailing, relative to no livelihood activity. In the same vein, with no livelihood as the reference, the probability of engaging in a vocation increases by about 12.30 percentage points if the woman’s household owns an enterprise. On the other hand, household enterprise ownership decreases the probability of choosing farming and paid employment by 1.79 and 16.72 percentage points respectively, relative to our benchmark category. We would like to emphasize that our study is arguably the first to include household enterprise ownership as a variable explaining livelihood activity choice.

Tables 6 presents the results, shown as marginal effects of the mlogit regression, that includes both “willingness to take risk” and religion as explanatory variables. By including the “willingness to take risk” variable, we can control for the effect risk preference has on livelihood choice. We can then isolate the effect of the social identity /social capital of religion on livelihood activity. Thus, the marginal effect of religion would refer to an effect it has on livelihood activity, other than its effect through risk behaviour-, that is the effect we hypothesize to work through religious social capital or identity.

**Table 6:** MLOGIT WITH BOTH WTR AND RELIGION. Multinomial Logit Marginal Effect (No Livelihood as Benchmark).

	VARIABLES	Farming	Petty Retailing	Vocation	Wage Employed
**Religion**	Pentecostal	0.1473***	−0.1695**	0.0296	0.0983
		(0.0332)	(0.0785)	(0.0623)	(0.0697)
	Catholic	0.1373***	−0.2368***	0.0675	0.1232*
		(0.0310)	(0.0870)	(0.0687)	(0.0718)
	Protestant	0.1472***	−0.2212***	0.0459	0.1353*
		(0.0335)	(0.0825)	(0.0649)	(0.0707)
	Muslim	−0.0073	−0.0835	−0.0135	0.1127
		(0.0107)	(0.1138)	(0.1020)	(0.0905)
**Risk Behaviour**	WTR	0.1470***	−0.0981	−0.00670	−0.0680**
		(0.0340)	(0.0667)	(0.0587)	(0.0343)
**Individual characteristics**	Age	0.0006	0.0160***	0.0033*	0.0043***
		(0.0005)	(0.0026)	(0.0019)	(0.0015)
	Married	0.00555	−0.0178	0.0260	0.0207
		(0.00760)	(0.0295)	(0.0237)	(0.0159)
	Education	0.0001	−0.1401***	0.0650	0.0347
		(0.00861)	(0.0470)	(0.0434)	(0.0320)
	General Social capital	−0.0007	−0.0268	0.0228	0.0051
		(0.0029)	(0.0175)	(0.0140)	(0.0093)
	Household head	0.0173**	0.0775*	0.0766***	−0.0029
		(0.0081)	(0.0415)	(0.0296)	(0.0222)
**Household characteristics**	Dependency ratio	0.0009	0.0918***	−0.0836***	−0.0053
		(0.0038)	(0.0220)	(0.0217)	(0.0121)
	Per-capita income	−0.0001	0.0004**	1.16e-05	0.0002***
		(6.84e-05)	(0.0002)	(0.0001)	(6.14e-05)
	Household farm	0.0356***	−0.0120	−0.0111	0.0012
		(0.0098)	(0.0322)	(0.0264)	(0.0196)
	Household enterprise	−0.0177***	0.3345***	0.1227**	−0.1666***
		(0.0050)	(0.0543)	(0.0493)	(0.0144)
	Observations	1209	1209	1209	1209

Note: Robust standard errors in parentheses; *** p < 0.01, ** p < 0.05, * p < 0.1.

We note that now that we include the variable “WTR” which shows the woman’s willingness to take risk, the marginal effects for Pentecostal, Catholic and Protestant are slightly higher for farming. This shows that the effect of risk is to decrease, to a small extent, the tendency of Pentecostals, Catholics, and Protestants relative to the non-religious of participating in farming. This makes sense since farming is a highly risky activity, and these religious groups are more risk averse than the non-religious (see Ayifah et al., 2020, as well as Table 4 in this current paper). The very significant positive coefficient on the WTR variable, however, shows that the more a woman is willing to take risk, the more likely she is to engage in farming^5^ . Thus with respect to farming, the effect of religion on farming operates through a strong positive religious social capital or identity effect, as well as a smaller negative effect through these groups aversion to risk and thus engaging in the risky act of farming.

In terms of Muslims, it seems there is no significant social identity and social capital effect happening. Any effect of risk aversion would seem to be too small to create a significant overall effect.

For Wage employed, while there is a statistically significant negative effect of the willingness to take risk variable on being wage employed, the magnitude of the marginal effects of religion remain very similar to before. Thus, the main effect of being Protestant or Catholic on being wage employed is a strong positive social capital or social identity effect.

Concerning petty retailing, the effect of willingness to take risk is insignificant, and thus the negative, largely unchanged marginal effects of being Protestant, Catholic and Pentecostal are due to a negative social capital and/or social identity effect. It seems that such social networking or clustering effects serves to keep women belonging to these religious groups away from petty retailing compared to the non-religious. Clearly petty retailing is considered the least sought after livelihood activity given that the non-religious women without associated social capital/identity have the highest probability of it as livelihood. Thus, having the advantage of religious social capital will encourage such individuals to cluster in more sought after livelihood activities, and discourage them from engaging in petty retailing.

### 4.2 Robustness tests

We tested the Independence of Irrelevant Alternatives (IIA) assumption using the Hausman specification tests (Hausman and McFadden, 1984), by comparing the general estimated model with a restricted model where we first eliminated (dropped) the different livelihood activity categories in turn from the livelihood activity choice-set to see if underlying livelihood activity choice behavior from the restricted livelihood activity choice-set obeys the IIA property. It is evident in all cases that IIA holds (Table 7, Table 8). Hence, we do not reject the multinomial logit specification.

**Table 7:** Hausman Tests of the independence of irrelevant alternatives (IIA): Religion no WTR.

Omitted Livelihood Activity	Chi2 values	Df	Prob > chi2	Evidence
1. No livelihood	1.83	7	0.9688	for Ho
2. Farming	−124.87	3	-----	for Ho
3. Petty retailing	−0.00	4	-----	for Ho
4. Vocation	−0.00	3	-----	for Ho
5. Paid employment	−0.00	3	-----	for Ho

**Note:** Ho: (Odds outcome I vs. outcome j) are independent of other alternatives.

If ch2 < 0, the estimated model does not meet the asymptotic assumptions of the test.

**Table 8:** Hausman Tests of the independence of irrelevant alternatives (IIA): Religion and WTR.

Omitted Livelihood Activity	Chi2 values	Df	Prob > chi2	Evidence
1. No livelihood	−61.48	5	------	for Ho
2. Farming	−212.09	29	------	for Ho
3. Petty retailing	9.09	18	0.9575	for Ho
4. Vocation	−112.95	4	------	for Ho
5. Paid employment	−172.75	12	-----	for Ho

**Note:** Ho: (Odds outcome I vs. outcome j) are independent of other alternatives.

If ch2 < 0, the estimated model does not meet the asymptotic assumptions of the test.

### 4.3 Limitations and Scope of the study

First, we restricted ourselves to the main livelihood activities relevant to the study area owing to data limitations and since it is difficult to provide an exhaustive list of all livelihood activities. Secondly, our study participants do not constitute a random sample of Ghanaian women as a whole, since our study participants came from only the Yilo and Manya Krobo Districts of the Eastern Region of Ghana. Therefore, we do not attempt to generalise our findings to the entire Ghanaian women population.

## 5 Conclusions and recommendation

In this paper, we investigated the effect of a woman’s religious affiliation on her choice of livelihood activity in the Yilo and lower Manya Krobo districts of Ghana. Our aim is to disentangle the various effects a woman’s religious affiliation could have on her choice of livelihood activity. The first effect relates to both social capital and social identity. The importance of religion as a specific type of social capital, and thus its particular role in choice of livelihood activity is investigated. Social capital is essential for facilitating and sustaining diverse income portfolios and access to opportunities and resources to individual households.

In addition, based on the ‘social identity theory’ in Social Psychology, it can be argued that having common religious beliefs can encourage individuals to group together. Such grouping can cause members of the same religion to engage in the same livelihood activities. The second effect we investigate is the effect of risk aversion resulting from belonging to various religious groups, which would then either encourage or discourage women belonging to these groups to or from engaging in certain livelihood activities.

When looking at the overall effect of religion on livelihood choice, we find that relative to non- religious women, Catholic, Pentecostal and Protestant women are more likely to be in farming, and less likely to be in petty retailing compared to being unemployed. Protestant women are also more likely to be wage employed than to be unemployed. However, the effect of being Muslim when it comes to livelihood activity is insignificant for women in our sample.

In general, women who are willing to take risk are more likely to choose farming, and less likely to be wage employed than they are of being unemployed.

The effect of religion on farming for Pentecostals, Catholics and Protestants is due to a very large social capital/social identity effect, and a smaller negative effect of risk aversion, because of belonging to these religious groups.

The effect of religion on being wage employed seems to result from a strong social identity or capital effect for Protestant and Catholic women. The effect of risk aversion because of belonging to these religious groups does not seem to have an effect on being wage employed.

For Petty retailing the effect of being Catholic, Pentecostal or Protestant results from a negative social capital/identity effect. Petty retailing is considered the least sought after livelihood activity in this area, and thus having the advantage of religious social capital will encourage such individuals to cluster in more sought after livelihood activities, and discourage them from engaging in petty retailing. Muslim women on the other hand, do not seem to derive any social capital or social identity benefits from their religion vis-à-vis their livelihood.

Thus, policies aimed at creating sustainable livelihoods and promoting employment for women in the Yilo and lower Manya Krobo districts of Ghana need to take into account the fact that belonging to certain religious groups provides an advantage in the way of social networking. It seems that non-religious women as well as Muslim women may be disadvantaged in this regard, especially with regards to farming and wage employment. In fact, the only livelihood activity that non-religious women appear to be able to engage in is petty retailing.

Policies also need to take into account the risk preferences of women in general with respect to promoting policies regarding farming and wage employment.

Our results suggest that other individual and household factors such as age, education, household headship, household farm ownership, and household enterprise ownership should also be considered in programmes and policy designs aimed at enhancing livelihood activities of women in this area.

Lastly, although it is generally believed that farming is the primary livelihood activity for most rural women, in the context of our current study we see that non-farm livelihood activities, especially petty retailing and vocational activities contribute significantly to livelihood activities of the women in our sample. Therefore, policies and programmes should ensure support to the non-farm sectors, at least in the context of our study area, especially petty retailing which more than half (52 percent) of the study, women depend on for a livelihood. In addition, to the extent that a sizeable number (20 percent approximately) of the women in our sample were not engaged in any livelihood activity, it shows the extent of unemployed women. There is therefore the need to implement programmes and policies aimed at creating sustainable livelihoods for these women.

## Funding

This study is based on research funded by a grant to the University of California, Davis from the Bill & Melinda Gates Foundation. The findings and conclusions contained within are those of the authors and do not necessarily reflect positions or policies of the Bill & Melinda Gates Foundation. We thank the iLiNS DYAD-G management team and the iLiNS Steering Committee for allowing us to use the data. Besides, we wish to acknowledge the work of the iLiNS SES Team especially Stephen Vosti, Travis Lybbert and Katherine Adams for the study design, the iLiNS DYAD-G SES enumerators for their work in the field, as well as, the data entry personnel. Lastly, we thank the women who participated in the study. All errors are those of the authors alone.

## Author statement

**Dr Aylit Romm**: Involved in conceptualisation, advising on methodology and analysis, writing up the paper.

**Dr Emmanuel Ayifah**: Funding acquisition, data curation, carrying out formal data analysis, paper review and editing.

**Professor Uma Kollamparambil**: Involved in conceptualisation, advising and supervising of methodology and data analysis, review and editing of paper.

## Footnotes

1 Dary and Kunnibe (2012), while not investigating the effect of religion specifically on livelihood activity choice, did include religion as control variables in their regression. The religion variables (which included only Muslims and Christians as a whole) were not significant. However, they simply looked at the probability of engaging in off farm activities, with no further breakdown of livelihood category. Religion itself was also not broken down in a more detailed way into specific Christian groups, as well as other religion (other than Muslim and Christian)

2 Stets and Burke (2000) posit that *social identity theory* emphasizes one’s identification or association with a particular group, while *identity theory* examines the roles or behaviors persons enact as members of a group.

3 See Ayifah et al. (2020) for a detailed account of how the risk aversion game worked.

4 Respondents were asked to indicate which of the following associations they belong to: Susu group, Farmer’s Association, Sewing group, Singing or music group, Church group, Informal retailers group, School committee, Development committee, Queen mothers’ association, Market association, Income earning group, Mother’s/Men’s support group

5 We acknowledge that there could be some endogeneity in the way of reverse causation with the WTR variable. While risk preference could, have an influence on the choice of livelihood activity, it is true in addition, that having a risky livelihood activity could induce a person to take less risk in other areas. However, in the case of farming, these two effects work in opposite directions. The effect from WTR to livelihood activity (farming) would be positive, while the effect from livelihood activity (farming) to WTR would be negative (see Bhandari and Kundu (2014) which shows evidence of such a negative effect). Thus, a positive coefficient on the WTR variable for farming may if anything be biased downwards. However, the fact that the coefficient is positive reflects the fact that there is definitely a positive effect running from WTR to farming.
